# Supplementary material for: Getting More Out of Biomedical Documents with GATE's Full Lifecycle Open Source Text Analytics
Source: PLoS Comput Biol. 2013 Feb 7;9(2):e1002854. doi: 10.1371/journal.pcbi.1002854 (PMC3567135; doi:10.1371/journal.pcbi.1002854)
Supplement: Dataset S1 — GATE software. Dataset S1 bundled with this paper contains a distribution of GATE (or see http://gate.ac.uk/download/). (TGZ) [file pcbi.1002854.s001.tgz › plos-gate/doc/27MonthReport.html]

GATE2 27-month report


## GATE 2: 27-month report

### Hamish Cunningham, Valentin Tablan, Cristian Ursu, Kalina Bontcheva, 1st October, 2001

This is the third in a series of progress reports for the GATE 2 project
(EPSRC grant GR/M31699, running from July 1999 to summer 2002).
The previous report is here.

Our work over the last 9 months, from January through September
2001 has centered on:

- releasing version 2 beta 1 of the system;- adapting GATE and the IE components delivered with it to Indic languages.

## GATE version 2

GATE version 2
has been completely redeveloped in Java, and is a stable, robust,
and scalable infrastructure for Natural Language Processing, which
allows users to focus on the NLP tasks, while mundane tasks like
data storage, format analysis, data visualisation are handled
by GATE. The new version has NLP components that will enable you
to reliably process documents, including Web documents supplied
as URLs, and obtain information such as the sentences they
contain, person names, organisations, etc. This is based on a set
of reusable NLP components, which you can also use outside GATE
by putting them into your own applications (e.g. a news indexing
service). GATE also provides standard tools for manual annotation
and performance evaluation, which are needed during application
development. GATE and its NLP modules have been successfully used
in a number of research projects and commercial applications.

## GATE and EMILLE

GATE has been upgraded as a result of the requirements of the
EMILLE project, and both the core system and the bundled Information
Extraction system are now proven capable of handling Indic (and many
other) languages.

The software now supports display of all the EMILLE languages that
are in the Unicode standard. This display is imperfect in JDK1.3 but
has been improved in JDK1.4. We are currently working on porting to
the latter system. In addition, the system supports input methods (for
editing of text) for 27 languages, including these EMILLE languages:
Bengali, Urdu, Hindi (two variants).

We have conducted successfull experiments in performing Named Entity
recognition in Bengali.

The work has been made available to the community under the GNU open
source library licence, and has already been taken up by the Max
Planck Institute technical group in Nijmegen, who have extended the
system's support for Chinese languages.
